# Supplementary material for: Sustainable power generation for at least one month from ambient humidity using unique nanofluidic diode
Source: Nat Commun. 2022 Jun 16;13:3484. doi: 10.1038/s41467-022-31067-z (PMC9203740; doi:10.1038/s41467-022-31067-z)
Supplement: Supplementary file 1 — Supplementary Information [file 41467_2022_31067_MOESM1_ESM.pdf]

Supplementary Information

Sustainable power generation for at least one month from ambient humidity using unique nanofluidic diode

Yong Zhang[1], Tingting Yang*[,1], Kedong Shang[1], Fengmei Guo[2], Yuanyuan Shang[2], Shulong Chang[2], Licong Cui[1], Xulei Lu[1], Zhongbao Jiang[1], Jian Zhou[1], Chunqiao Fu[1], Qi-Chang He*[,1,3]

[1]Tribology Research Institute, School of Mechanical Engineering, Southwest Jiaotong University, Chengdu 610031, PR China
[2]Key Laboratory of Material Physics, Ministry of Education, School of Physics and Microelectronics, Zhengzhou University, Zhengzhou 450052, PR China
[3]MSME, Univ Gustave Eiffel, CNRS UMR 8208, F-77454 Marne-la-Vallée, France

*Corresponding authors. Email addresses: tingtingyang@swjtu.edu.cn; qi-chang.he@u-pem.fr

This PDF file includes:

Supplementary Notes 1-3;

Supplementary Methods;

Supplementary Table 1-4;

Supplementary Figure 1-19.

Supplementary References.

Supplementary Notes

1 Analysis of built-in potential V_B in ionic diode–type AAO/CNT junction

Carriers in semiconductors and ions in solution are similar in many ways. In nanopore, CNT with negative surface charge can be regarded as P-type semiconductor, and AAO with positive surface charge can be regarded as N-type semiconductor. Therefore, the nanopore diode has a rectification effect similar to the semiconductor PN junction. Analogous to the classic thermodynamic model of P-N semiconductor junction, the built-in potential V_B in ionic diode–type AAO/CNT junction is derived as follows [1,2]

In a humid environment, CNT is expected to freely dissociate hydrogen ions to form a weak acid environment, and its surface is negatively charged. In a weak acid environment, due to the amphoteric -OH groups, the surface polarity of AAO channel is positively charged. This dissociation can be described by the following chemical equilibria:

$CNT(COOH) \iff H^{+}+CNT(COO^{-})$ Eq. 1.1

$AAO(OH)+H_2O \iff AAO(OH_2^{+})+OH^{-}$ Eq. 1.2

CNT (COO^-) results from CNT dissociates hydrogen ions, and its surface leaves fixed COO^-, AAO (OH^{2+}) comes from AAO dissociates OH^-, and its surface remains fixed OH_2^+. When the degree of ionization is not too large, the initial concentrations of [COO^-] and [H^+] in the CNT domain can be written as

$$[H^+]^{\mathrm{CNT}} \approx \sqrt{K_{\mathrm{CNT}}[\mathrm{CNT}]},\ [COO^-]^{\mathrm{CNT}} \approx \sqrt{K_{\mathrm{CNT}}[\mathrm{CNT}]} \qquad \text{Eq. 1.3}$$

where [X] represents the initial concentrations of substance X, and K_X denotes the dissociation coefficient of substance X. Similarly, the initial concentrations of [OH_2^+] and [OH^-] in the AAO domain can be written as

$$[OH_2^+]^{\mathrm{AAO}} \approx \sqrt{K_{\mathrm{AAO}}[AAO]},\ [OH^-]^{\mathrm{AAO}} \approx \sqrt{K_{\mathrm{AAO}}[AAO]} \qquad \text{Eq. 1.4}$$

After joining CNT and AAO, the freely dissociated H^+ mobile cations from the CNT diffuse to the AAO while the OH^- anions from the AAO close to the interface diffuse to the CNT, resulting in the formation of an ionic double layer (IDL) with dissociation constant K_{IDL}. Such IDL is similar to the depletion layer at a P-N semiconductor junction:

$IDL \iff H^{+}+OH^{-}$ Eq. 1.5

The diffusion current density can be expressed as

$$J_{\mathrm{Diff}} = qD\frac{dC(x)}{dx} \qquad \text{Eq. 1.6}$$

where q, D, C(x), x represent, respectively, the carrier charge, diffusion coefficient, concentration and the position along the direction perpendicular to the CNT/AAO interface.

Diffusion of H^+ and OH^- leaves behind excess fixed COO^- and fixed OH_2^+ charges on the CNT and AAO side, respectively, yielding a built-in electric field E directed from AAO to CNT. The electric field counterbalances the charge diffusion and yields drift current:

$$J_{\mathrm{Drift}} = q\mu C(x)E(x) \qquad \text{Eq. 1.7}$$

where μ represents the mobility.

The net current is zero at equilibrium (open-circuit) state, described by the Nernst Planck equation:

$$J = J_{\mathrm{Drift}} - J_{\mathrm{Diff}} = q\mu C(x)E(x) - qD\frac{dC(x)}{dx} = 0 \qquad \text{Eq. 1.8}$$

Since E(x)=-dV/dx, Eq.1.8 can be written as Eq. 1.9 after using Einstein's relation μ/D=$q/K_{\mathrm{B}}T$:

$$-\frac{q}{K_B T}\frac{dV(x)}{dx} = \frac{1}{C(x)}\frac{dC(x)}{dx} \qquad \text{Eq. 1.9}$$

Eq. 1.10 is obtained after integration of Eq. 1.9 with the boundary conditions.

$$-\frac{q}{K_{\mathrm{B}}T}\int_{V_{CNT}}^{V_{AAO}} dV = \int_{[H^+]^{AAO}}^{[H^+]^{CNT}} \frac{1}{C}dC \quad (\mathrm{H^+\ cation})$$

$$-\frac{q}{K_{\mathrm{B}}T}\int_{V_{CNT}}^{V_{AAO}} dV = \int_{[OH^-]^{CNT}}^{[OH^-]^{AAO}} \frac{1}{C}dC \quad (\mathrm{OH^-\ anion}) \qquad \text{Eq. 1.10}$$

At equilibrium (open-circuit) state, the built-in potential V_{B} is equal to V_{AAO}-V_{CNT}:

$$V_B = \frac{K_B T}{q} ln\frac{[H^+]^{CNT}}{[H^+]^{AAO}} = \frac{K_B T}{q} ln\frac{[OH^-]^{AAO}}{[OH^-]^{CNT}} \qquad \text{Eq. 1.11}$$

According to Eq. 1.11, Eq. 1.12 can be deduced as

$$\frac{[H^+]^{CNT}}{[H^+]^{AAO}} = \frac{[OH^-]^{AAO}}{[OH^-]^{CNT}} \qquad \text{Eq. 1.12}$$

It is assumed that a small amount of IDL is generated at the CNT/AAO interface, leading to a low concentration of [IDL] in both CNT and AAO:

$$[H^+]^{CNT}[OH^-]^{CNT} = [H^+]^{AAO}[OH^-]^{AAO} = K_{IDL}[IDL] \qquad \text{Eq. 1.13}$$

According to Eq. 1.3, Eq. 1.4 and Eq. 1.13, the initial concentration of minority free ions can be estimated as follows:

$$[OH^-]^{\mathrm{CNT}} = \frac{K_{IDL}[IDL]}{[\mathrm{H}^+]^{CNT}} = \frac{K_{IDL}[IDL]}{\sqrt{\mathrm{K_{CNT}[CNT]}}}$$

$$[H^+]^{\mathrm{AAO}} = \frac{K_{IDL}[IDL]}{[OH^-]^{AAO}} = \frac{K_{IDL}[IDL]}{\sqrt{K_{\mathrm{AAO}}[AAO]}} \qquad \text{Eq. 1.14}$$

Then Eq. 1.11 V_{B} can be re-written as

$$V_{\mathrm{B}} = \frac{K_B T}{q} ln\frac{\sqrt{\mathrm{K_{AAO}[AAO]}}\sqrt{\mathrm{K_{CNT}[CNT]}}}{K_{IDL}[IDL]} = \frac{K_B T}{q}\left(ln\frac{\sqrt{\mathrm{K_{AAO}K_{CNT}}}}{K_{IDL}} + ln\frac{\sqrt{\mathrm{[CNT][AAO]}}}{[IDL]}\right) \qquad \text{Eq. 1.15}$$

2 Analysis of surface charge density of AAO and CNT

The surface charge density σ on the AAO and CNT is calculated from Zeta potential (ζ) as follows[3,4]:

$$\sigma(\varphi_d) = \frac{2\varepsilon\varepsilon_0\kappa}{\beta e}\left[\sinh\frac{\beta e\varphi_d}{2} + \frac{2}{\kappa a}\tanh\frac{\beta e\varphi_d}{4}\right] \qquad \text{Eq. 2.1}$$

Here, $\varepsilon\varepsilon_0$ is the permittivity, the curvature of radius and κ^{-1} the Debye screening length given by $\kappa^2=\beta e^2 n/\varepsilon\varepsilon_0$, with n the ion concentration and $\beta^{-1}=K_B T$. In addition, φ_d is the diffuse layer potential proportional to Zeta potential (ζ) which usually denotes the potential difference between the inner immobile layer and the bulk of solution. Since σ increases monotonously with φ_d, after plasma surface treatment, the increased ζ leads to an increased surface charge density σ. More surface charge means larger initial functional group concentrations of [CNT] and [AAO]. According to Eq. 1.15 in the supplementary materials, the value of the built-in potential V_B thus also increases.

3 Analysis of open circuit voltage and short circuit current in the HEEG

V_{OC}, V_B, and V_{redox} represent the measured open-circuit voltage, the built-in potential of the hybrid membrane, and the redox potential. The measured open-circuit voltage (V_{OC}) actually consists of two parts: the built-in potential (V_B) that is generated by the power source and the redox potential (V_{redox}) that is produced by the unequal potential drop at the electrode-solution interface, which satisfy the Nernst equation:

$$V_{OC}=V_B+V_{redox} \qquad \text{Eq. 3.1}$$

$$V_{redox}=V^0+\frac{RT}{nF}\ln\frac{\Pi a^{v}_{\text{Reactant}}}{\Pi a^{v'}_{\text{product}}} \qquad \text{Eq. 3.2}$$

V^0 is standard electromotive force for redox reactions, v and v' are the stoichiometric numbers of reactants and products. n is the number of electrons participating in the reaction. R, T and F are the molar gas constant, temperature, and Faraday constant, respectively. a_{Reactant}, a_{product} is the activity, and the physical meaning of the activity is the effective concentration. Reactant refers to the bottom electrode metal, O_2, H_2O, *etc*. The product contains metal oxides, metal hydroxides, hydroxyl metal complex ions, *etc*.

The short-circuit current is positively related to the electrochemical reaction rate of the electrode. According to chemical kinetics, the relationship between the reaction rate v and the reaction activation energy ΔG is

$$v=kc\exp\left(-\frac{\Delta G}{RT}\right) \qquad \text{Eq. 3.3}$$

where k is the pre-exponential factor and c is the reactive particle concentration.

Supplementary Methods

Numerical Simulation

The spatial distribution profiles of electric field and the concentration of mobile ions are both theoretically studied using a commercial finite-element software package COMSOL (version 5.6) Multiphysics based on the coupled two-dimensional Nernst-Planck-Poisson (PNP) equations. The Nernst-Planck-Poisson (PNP) equations are shown below:

$$j_i=D_i\left(\nabla c_i+\frac{z_i F c_i}{RT}\nabla\varphi\right) \qquad \text{Eq 4.1}$$

$$\nabla^2\varphi = -\frac{F}{\varepsilon}\sum z_i c_i \qquad \text{Eq 4.2}$$

$$\nabla \cdot j_i = 0 \qquad \text{Eq 4.3}$$

where, j_i, D_i, c_i, z_i, φ, ε, F, R, and T are the ionic flux, diffusion coefficient, ion concentration, valence number for each species i, electrical potential, dielectric constant of the electrolyte solution, Faraday constant, universal gas constant, and absolute temperature, respectively.

The fluxes of mobile ions and transport properties of a charged nanopore are described by Equation 4.1 which is the Nernst-Planck equation. The relationship between the electrical potential and ion concentrations is defined by Equation 4.2 which is the Poisson equation. When the system reaches a stationary regime, the time-independent continuity Equation 4.3 should be satisfied by the flux. With the given geometry and suitable boundary conditions, the coupled PNP equations could be solved with finite-element calculations for the ion concentration distribution and the electric field distribution. The system is generally simplified by assuming steady-state conditions. And in order to simplify the calculation process, the numerical simulated model based on 2D structure is shown in the following figure. It contains a 3000 nm long AAO channel (pore size: 90 nm) and a 1000 nm long CNT channel (pore size: 50 nm). Two water reservoirs whose size is 200×500 nm^2 are introduced to set the practical environment in COMSOL. The ion flux has the zero normal components at boundaries:

$$n \cdot j_i = 0 \qquad \text{Eq 4.4}$$

The boundary condition for the potential φ on the channel walls is:

$$n \cdot \nabla\varphi = -\frac{\sigma}{\varepsilon} \qquad \text{Eq 4.5}$$

where, σ represents the surface charge density. It's worth noting that the surface charge density of the AAO channel is set to be $+5e^{-5}$ C·m^{-2} . And the surface charge density of CNT channel ($-1.5e^{-4}$ C·m^{-2}) is set to three times larger than the surface charge density of AAO channel according to the zeta potential. For the ionic rectification simulation, the initial concentration of the mobile ions (H^+ and OH^-) in the left reservoir is set to 10^{-10} mol/L and the right is set to 10^{-7} mol/L. The applied potential is 20 V.

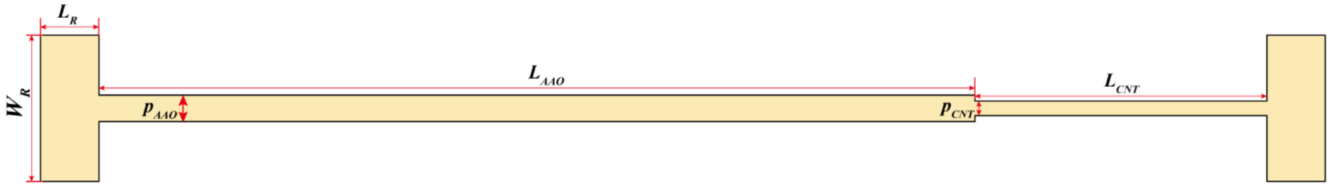

Supplementary Table 1| Performance parameter comparison of HEEGs at RH environment

Material	RH/Δ RH(%)	V_{OC} Voltage (V)	I_{SC} Current (nA)	J_{SC} Current density (uA·cm^{-2})	Output power density (μW·cm^{-2})	Continuous V_{OC} working time	Continuous I_{SC} working time	Ref
**Protein nanowire**	50	0.5	250	17	5 ($\frac{1}{4}V_{OC}\cdot J_{SC}$)	1500 h	20 h	7
**Graphene oxide bulk**	85	0.45	/	0.9	2.02 (Power on load)	100 h	30-50 s	8
**Gradient graphene oxide and graphene oxide**	80	1.50	98-136	/	32 mW·cm^{-3} (Power on load)	10 min	10 min	9
**Graphene oxide and sodium polyacrylate**	80	0.60	>100	>1	0.07 (Power on load)	120 h	<1 min	10
**Graphene oxide film**	70	0.4-0.7	/	2-25	18 ($V_{OC}\cdot J_{SC}$)	200 s	200 s	11
**TiO_2 nanowire**	85	0.52	/	8.0	4.16 ($V_{OC}\cdot J_{SC}$)	400 s	400 s	12
**Polyelectrolyte membrane (PSSA)**	Ambient RH	0.8	100000	100	17 (Power on load)	1400 s	48 h	13
**Biological nanofiber**	99	0.11	25	0.025	0.63 nW·cm^{-2} (Power on load)	168 h	/	14
**Gradient polypyrrole foam**	85	0.06	/	10	0.69 ($V_{OC}\cdot J_{SC}$)	14 s	14 s	15
**Paper**	70	0.25	15	0.01	/	<600 s	<600 s	16
**Bilayer of polyelectrolyte film**	85	1.38	950	4.00	5.52 μW·cm^{-2} ($V_{OC}\cdot J_{SC}$)	258 h	150 h	17
**Gradient cellulose membrane**	82	0.83	533	5.93	4.92 ($V_{OC}\cdot J_{SC}$)	12 h	12 h	18
**HEEG**	**93**	**1.1**	**7700**	**11.3**	**12.43 μW·cm^{-2} ($V_{OC}\cdot J_{SC}$) 1.3 μW·cm^{-2} 277 μW·cm^{-3} (Power on load)**	**at least 720 h**	**at least 785 h**	**This work**

Supplementary Table 2| elements content variation of different treating condition for CNT film and AAO membrane.

Plasma treating method of CNT	O/C (%)
Without plasma treating	8.02
With plasma treating	34.36
Treating after 7 days	24.17

Plasma treating method of AAO	Al/O (%)
Without plasma treating	46.16
With plasma treating	46.93
Treating after 7 days	44.59

Supplementary Table 3| Standard electromotive force of different metals.

Electrode reaction	φ^0/V
Al^{3+}+3e^-=Al	-1.66
Ti^{2+}+2e^-=Ti	-1.63
Zn^{2+}+2e^-=Zn	-0.763
Ga^{3+}+3e^-=Ga	-0.549
Ni^{2+}+2e^-=Ni	-0.257
Mo^{3+}+3e^-=Mo	-0.22
Au^{3+}+3e^-=Au	1.5

Supplementary Table 4| Relative humidity values corresponding to different kinds of saturated salt solutions (Measured at laboratory temperature).

Saturated salt solution	RH (%)
Lithium Chloride	11
Potassium Carbonate	40
Sodium chloride	70
Potassium Sulfate	93

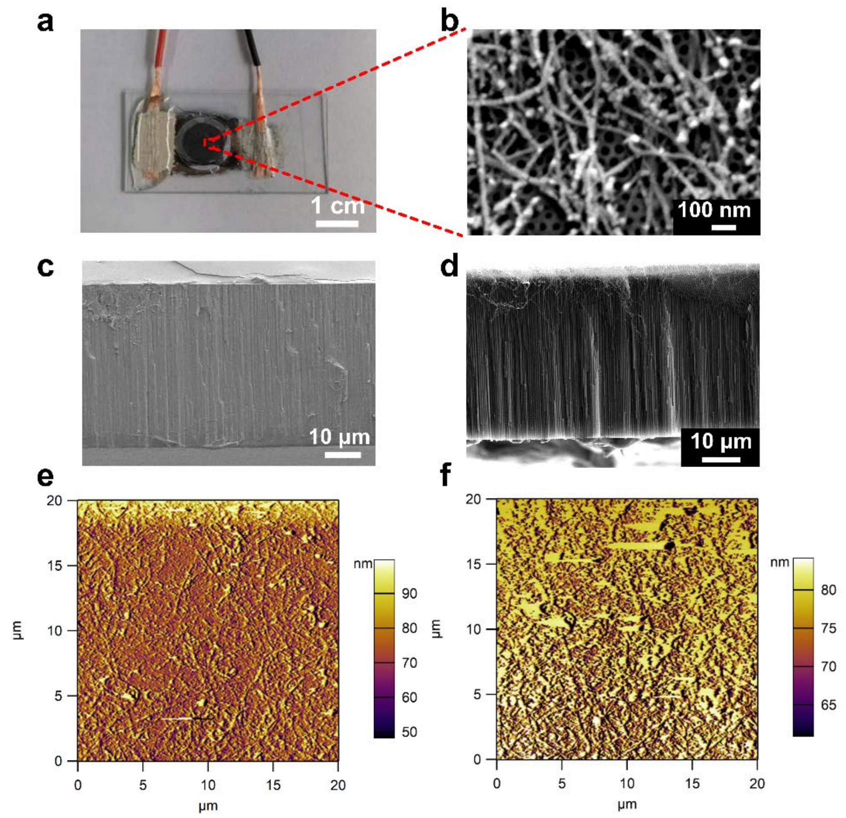

Supplementary Figure 1. a) Optical image of HEEG. b) SEM image of porous carbon nanotube networks, water molecular can easily absorbs on the CNT network skeleton and dissociates into anions and cations. The AAO nanopores under the CNT network can be clearly seen. c) and d) Cross-sectional SEM images of AAO membrane with and without CNT attached, which show a regular and straight nanochannels that facilitate charged ions to get into and pass through. e) The topography of the top surface area of the device swept out without moisture action (21.8°C, 38% RH) and f) The topography of the top surface area of the device swept out after moisture action (21.8°C, 60% RH). Although the

experimental results have proved that liquid water does exist on the surface of the device morphology in a humid environment, it is still unknown whether the Debye screening effect plays a role in power generation. However, since it is common to introduce the concept of Debye screening length in the research dealing with pore size, we still use this concept of Debye screening effect in the manuscript.

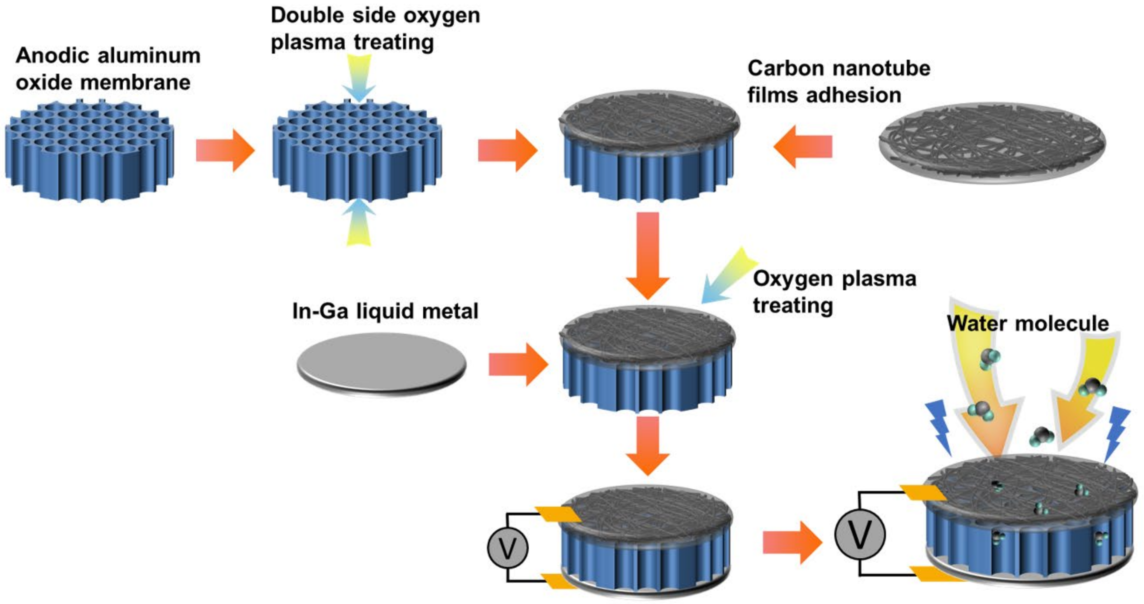

Supplementary Figure 2. The process of HEEG fabrication. Detailed process is illustrated in the method part.

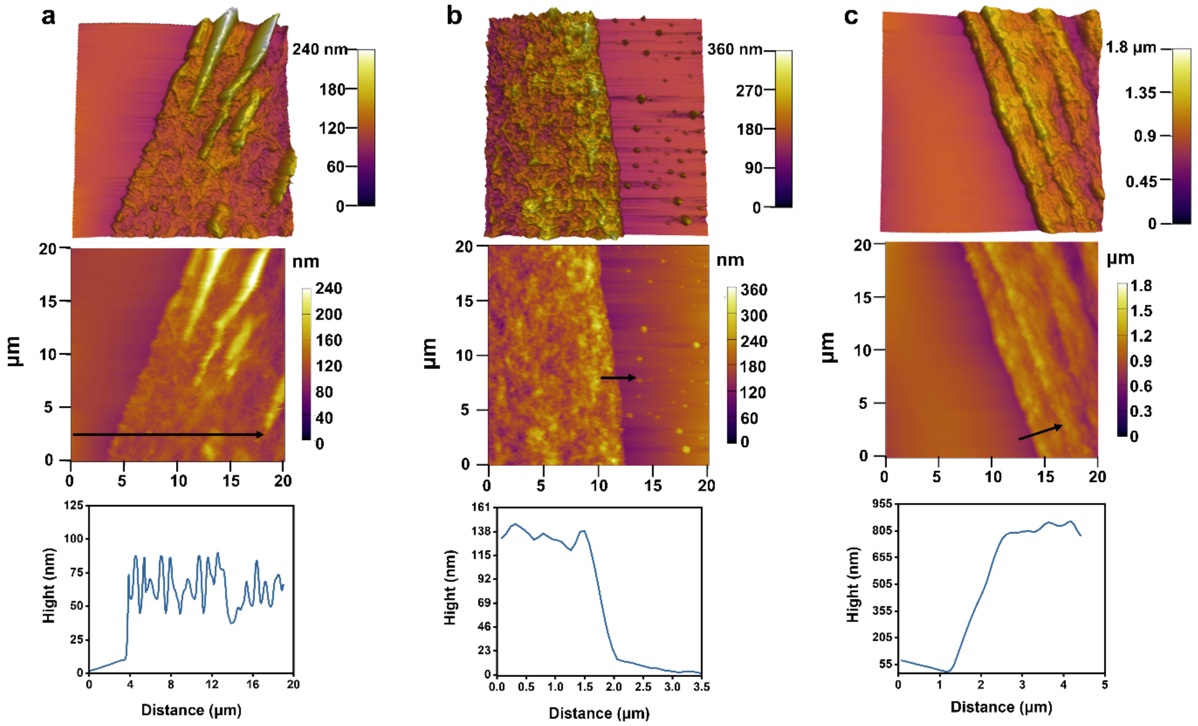

Supplementary Figure 3. AFM characterization of CNT thickness. a) b) c) are CNT films with different thickness. From top to bottom, they are three-dimensional, two-dimensional and height curves. The black arrow in the two-dimensional image represents the scan direction and path. Average thickness for different CNT films: a. 50~70 nm, b. 120~140 nm, c. ~800 nm.

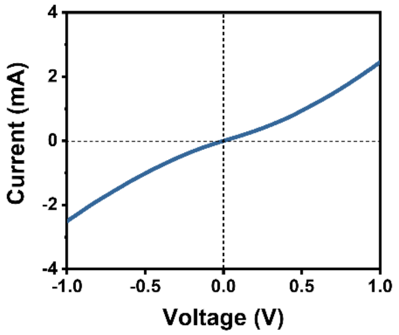

Supplementary Figure 4. The square resistance of CNT film, test volume ~1 cm × 1 cm × 140 nm.

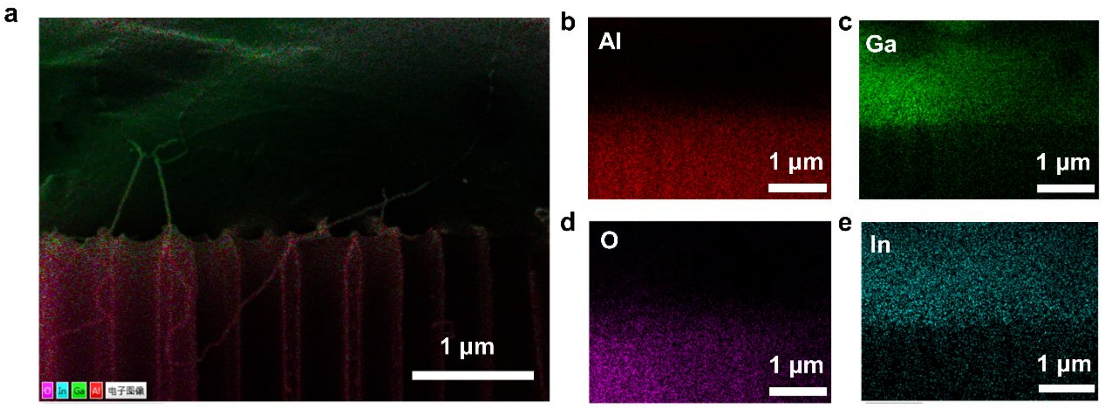

Supplementary Figure 5. Cross-sectional energy dispersive spectroscopy (EDS) mapping of In-Ga liquid metal and AAO interface.

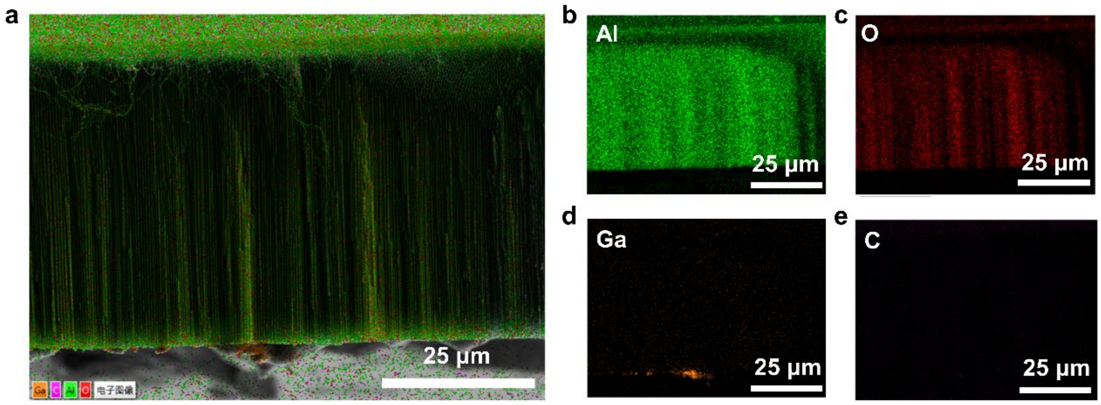

Supplementary Figure 6. Cross-sectional EDS mapping for whole sandwiched structure of CNT, AAO and In-Ga.

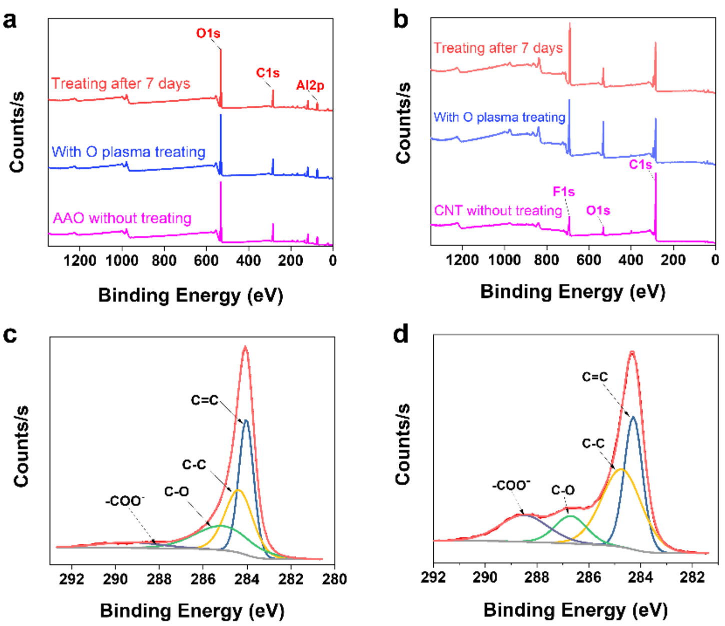

Supplementary Figure 7. The XPS spectra of a) AAO membrane and b) CNT film show the changes of the content of different elements for different treatments. c) d) Fine spectroscopy of XPS for CNT before and after plasma treating, which demonstrates that O-plasma treating can enhance the oxygen functional groups content.

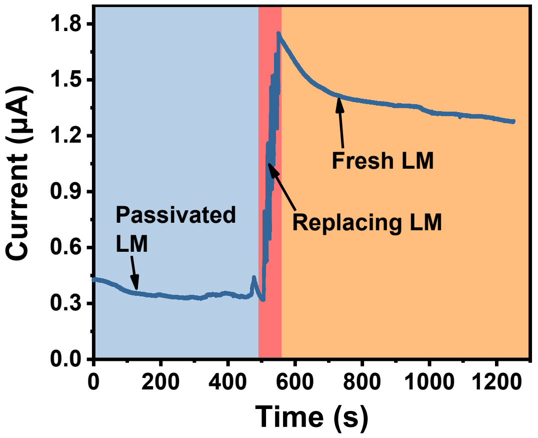

Supplementary Figure 8. By replacing the original passivated liquid metal (LM) with the device after 24 hours of working, the current performance of the device has been greatly improved.

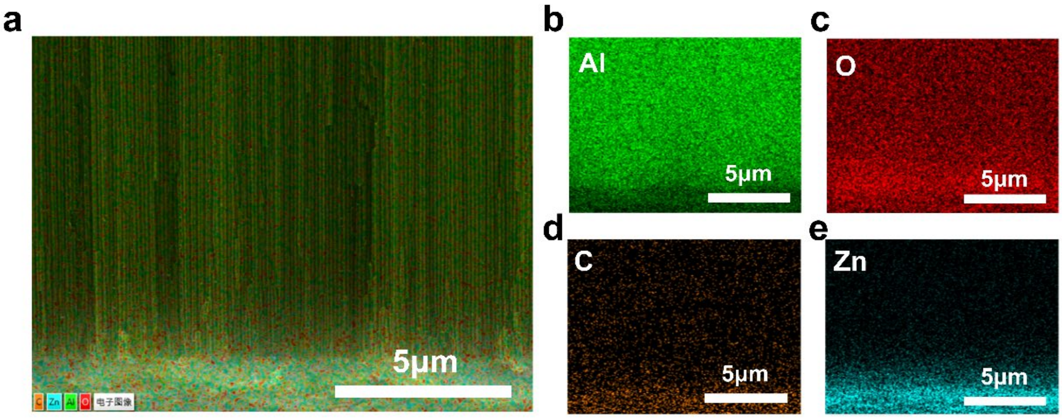

Supplementary Figure 9. After 25 days of continuous power generation, the element distribution map of the Zn-AAO cross-section shows that the zinc element has a tendency to diffuse towards the top electrode.

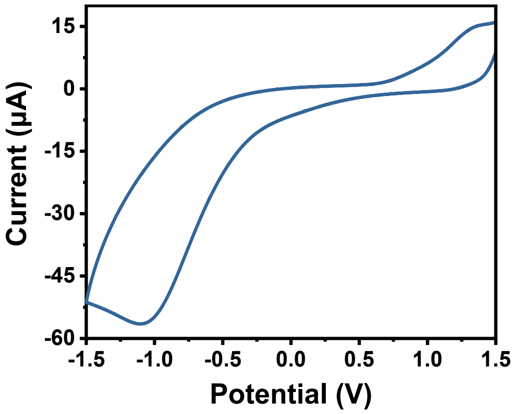

Supplementary Figure 10. The volt-ampere characteristic curve, the device is placed in a 93% RH humidity environment, and the window voltage is -1.5 V to 1.5 V.

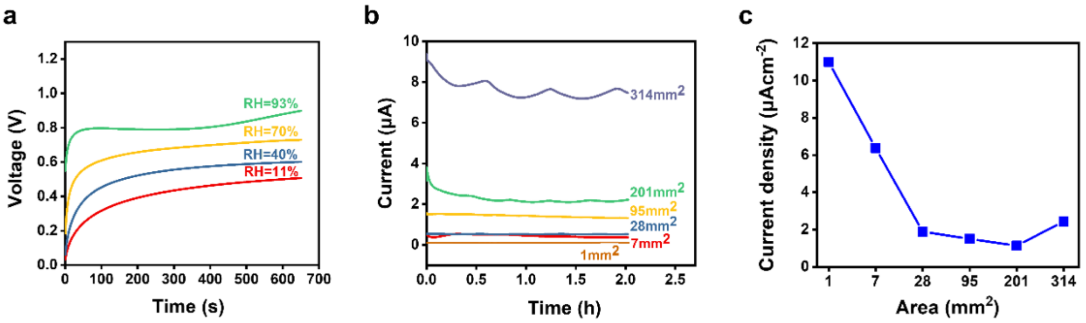

Supplementary Figure 11. a) Voltage changes with time for different relative humidity (working area 95 mm^2). b) The current value changes with time for different working area. c) Current density corresponding to different device area, when working area of 1 mm^2 has the highest value 11.3 $\mu A\cdot cm^{-2}$.

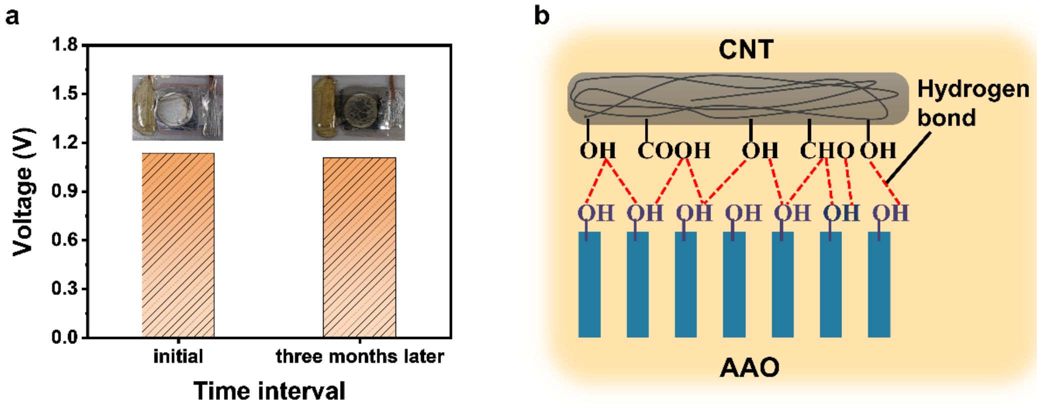

Supplementary Figure 12. a) The degradation of open circuit voltage of power generation devices after being placed in different environment for 90 days totally (93% high humidity condition for 30 days and ambient condition for 60 days). b) Schematic diagram of the hydrogen bond connection between AAO and CNT to enhance the structural stability of the hybrid film.

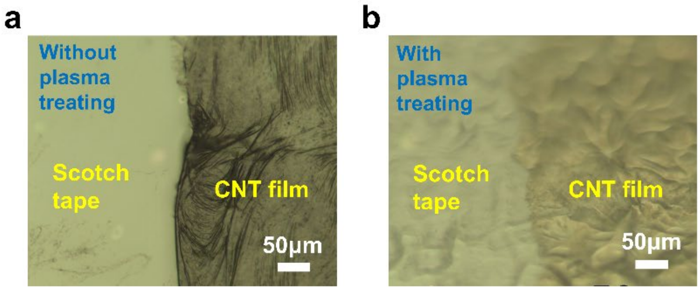

Supplementary Figure 13. a) and b) are the CNT films that were peeled off from the devices without plasma treating and with plasma treating using scotch tape, respectively. In Figure a, the color of the CNT film is darker, indicating that the bonding force of the AAO-CNT interface is weaker; the color of the CNT film in Figure b is lighter, indicating that the bonding force of the AAO-CNT interface is stronger.

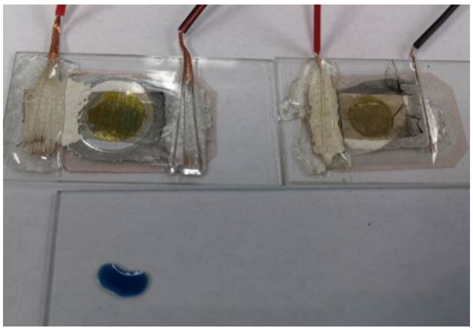

Supplementary Figure 14. The blue Bromothymol Blue indicator was dropped on the surface of the device and gradually turned yellow after ~8 minutes, indicating that the surface of the device was weakly acidic.

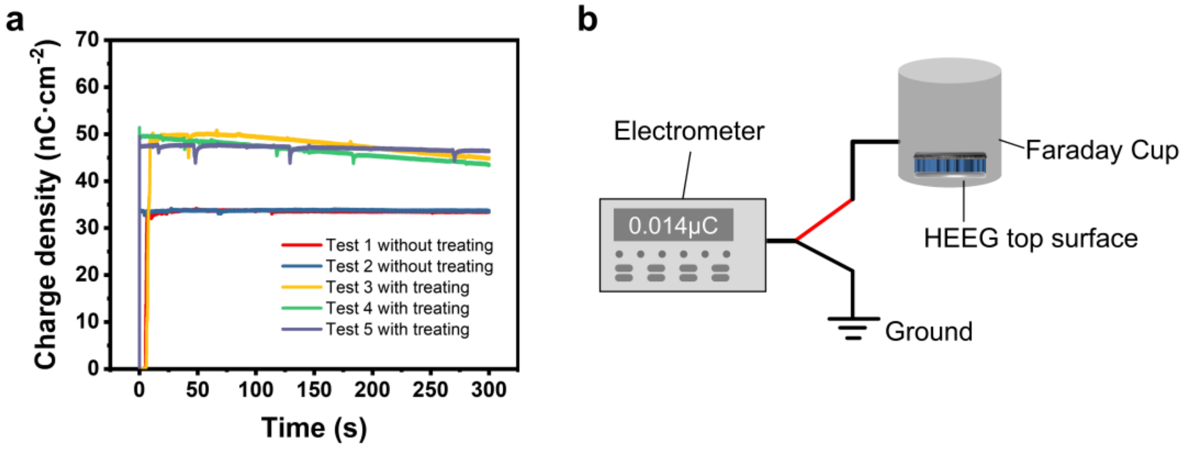

Supplementary Figure 15. Test of the amount of charge on the top surface of the device under open circuit condition (60% RH). a) the Faraday cup and electrometer are used to test the surface charge of the top surface of HEEG. After the device is treated with oxygen plasma, its surface charge density is improved to a certain extent; b) a schematic diagram of the wiring during the test process. During the test, the top surface of the device is close to the surface of the metal cup.

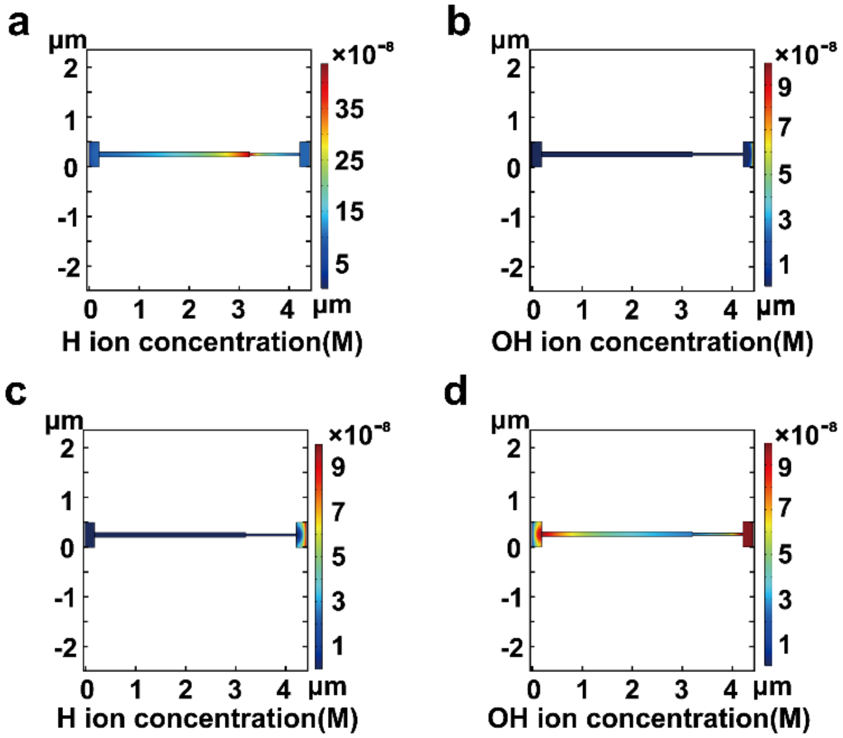

Supplementary Figure 16. a). The concentration distribution of H^+ under positive bias, H^+ is concentrated near the junction. b). The concentration distribution of OH^- under positive bias, OH^- is concentrated in the CNT film. c) H^+ concentration distribution under the action of reverse bias voltage, H^+ is concentrated on the side of the CNT film. d) The concentration distribution of OH^- ions under the action of reverse bias, OH^- is transported towards the bottom electrode.

For the ionic rectification simulation, the initial concentration of the mobile ions (H^+ and OH^-) in the left reservoir is set to 10^{-10} mol/L and the right is set to 10^{-7} mol/L. The applied potential amplitude is 20 V.

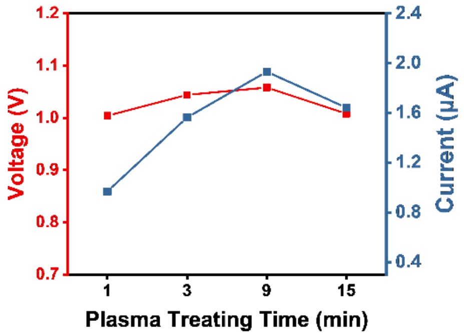

Supplementary Figure 17. Influence of plasma treating time to device's V_{OC} and I_{SC}.

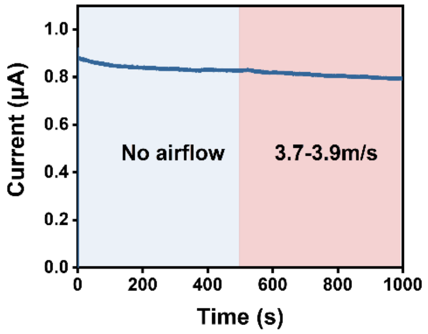

Supplementary Figure 18. Current performance image of HEEG with and without airflow interference, which shows that airflow has no effects on HEEG current performance.

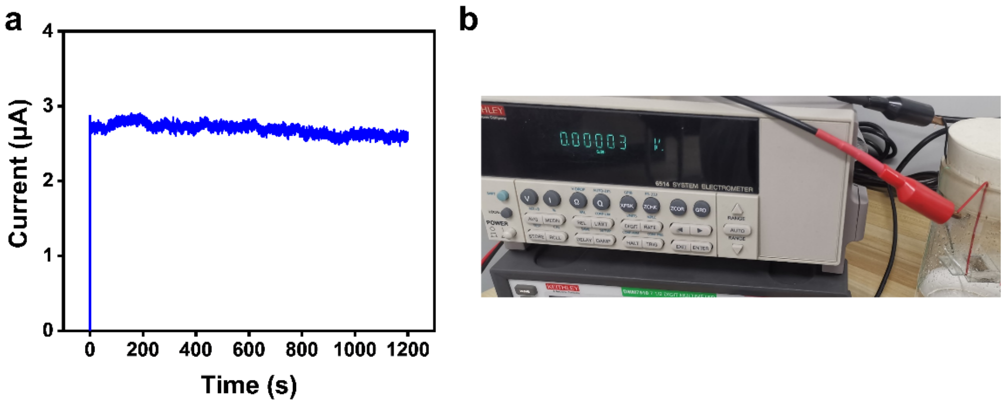

Supplementary Figure 19. Test of the electronic output performance of "CNT-LM" structure. a) the current signal produced by the CNT-LM structure; b) a photo of the captured voltage signal showing

0 V.

Supplementary References

1. Streetman, B. G. & Banerjee, S. Solid state electronic devices. Vol. 10 (Pearson/Prentice Hall Upper Saddle River, NJ, 2006).
2. Kim, H. J., Chen, B., Suo, Z. & Hayward, R. C. Ionoelastomer junctions between polymer networks of fixed anions and cations. *Science* **367**, 773-776 (2020).
3. Behrens, S. H. & Grier, D. G. The charge of glass and silica surfaces. *J. Chem. Phys.* **115**, 6716-6721 (2001).
4. Qin, Y. et al. Constant Electricity Generation in Nanostructured Silicon by Evaporation-Driven Water Flow. *Angew. Chem. Int. Ed.* **59**, 10619-10625 (2020).
5. Li, L. et al. A novel, flexible dual-mode power generator adapted for wide dynamic range of the aqueous salinity. *Nano Energy* **85**, 105970 (2021).
6. Jia, Y. et al. Nanotube-Silicon Heterojunction Solar Cells. *Adv. Mater*. **20**, 4594-4598 (2008).
7. Liu, X. M. et al. Power generation from ambient humidity using protein nanowires. *Nature* **578**, 550–554 (2020).
8. Cheng, H. H. et al. Spontaneous power source in ambient air of a well-directionally reduced graphene oxide bulk. *Energy Environ. Sci.* **11**, 2839–2845 (2018).
9. Huang, Y. X. et al. Interface-mediated hygroelectric generator with an output voltage approaching 1.5 volts. *Nat. Commun*. **9**, 4166 (2018).
10. Huang, Y. X. et al. All-region-applicable, continuous power supply of graphene oxide composite. *Energy Environ. Sci.* **12**, 1848–1856 (2019).
11. Xu, T. et al. Electric power generation through the direct interaction of pristine graphene-oxide with water molecules. *Small* **14**, 1704473 (2018).
12. Daozhi Shen. et al. Self-Powered Wearable Electronics Based on Moisture Enabled Electricity Generation. *Adv. Mater.* **30**, 1705925 (2018)
13. Xu, T. et al. An efficient polymer moist-electric generator. *Energy Environ. Sci.* **12**, 972-978 (2019).
14. Li, M. J. et al. Biological nanofbrous generator for electricity harvest from moist air flow. *Adv. Funct. Mater.* **29**, 1901798 (2019).
15. Xue, J. L. et al. Vapor-activated power generation on conductive polymer. *Adv. Funct. Mater*. **26**, 8784–8792 (2016).
16. Gao, X. et al. Electric power generation by paper materials. *J. Mater. Chem. A.* **7**, 20574–20578 (2019).
17. Wang, H., Sun, Y., He, T. et al. Bilayer of polyelectrolyte films for spontaneous power generation in air up to an integrated 1,000 V output. *Nat. Nanotechnol.* **16**, 811–819 (2021).
18. Lee, S., Eun, J. & Jeon, S. Facile fabrication of a highly efficient moisture-driven power generator using laser-induced graphitization under ambient conditions. *Nano Energy* **68**, 104364 (2020).
